# Self-reported halitosis and emotional state: impact on oral conditions and treatments

**DOI:** 10.1186/1477-7525-8-34

**Published:** 2010-03-26

**Authors:** Salvatore Settineri, Carmela Mento, Simona C Gugliotta, Ambra Saitta, Antonella Terranova, Giuseppe Trimarchi, Domenico Mallamace

**Affiliations:** 1 Department of Neuroscience, Psychiatry and Anaesthesiology, University of Messina, Via Consolare Valeria, 1, 98100 Messina, Italy; 2 Department of Odontostomatology, University of Messina, Italy; 3 SEFISTAT, Department of Economic, Financial, Social, Environmental, Statistical and Territorial Sciences, University of Messina, Italy

## Abstract

**Background:**

Halitosis represents a common dental condition, although sufferers are often not conscious of it. The aim of this study was to examine behavior in a sample of Italian subjects with reference to self-reported halitosis and emotional state, and specifically the presence of dental anxiety.

**Methods:**

The study was performed on Italian subjects (N = 1052; range 15-65 years). A self-report questionnaire was used to detect self-reported halitosis and other variables possibly linked to it (sociodemographic data, medical and dental history, oral hygiene, and others), and a dental anxiety scale (DAS) divided into two subscales that explore a patient's dental anxiety and dental anxiety concerning dentist-patient relations. Associations between self-reported halitosis and the abovementioned variables were examined using multiple logistic regression analysis. Correlations between the two groups, with self-perceived halitosis and without, were also investigated with dental anxiety and with the importance attributed to one's own mouth and that of others.

**Results:**

The rate of self-reported halitosis was 19.39%. The factors linked with halitosis were: anxiety regarding dentist patient relations (relational dental anxiety) (OR = 1.04, CI = 1.01-1.07), alcohol consumption (OR = 0.47, CI = 0.34-0.66), gum diseases (OR = 0.39, CI = 0.27-0.55), age > 30 years (OR = 1.01, CI = 1.00-1.02), female gender (OR = 0.71, CI = 0.51-0.98), poor oral hygiene (OR = 0.65, CI = 0.43-0.98), general anxiety (OR = 0.66, CI = 0.49-0.90), and urinary system pathologies (OR = 0.46, CI = 0.30-0.70). Other findings emerged concerning average differences between subjects with or without self-perceived halitosis, dental anxiety and the importance attributed to one's own mouth and that of others.

**Conclusions:**

Halitosis requires professional care not only by dentists, but also psychological support as it is a problem that leads to avoidance behaviors and thereby limits relationships. It is also linked to poor self care. In the study population, poor oral health related to self-reported halitosis was associated with dental anxiety factors.

## Background

Halitosis is a term used to describe oral malodor and is a common reason for seeking professional dental care. Some studies have estimated the prevalence of halitosis to be between 22% and 50%, others between 6% and 23% [1,2]. According to the American Dental Association, 50% of the adult population have suffered from an occasional oral malodor disorder, while 25% appear to have a chronic problem. As a result, there has been an increase in dentist consultations and in commercial business interests in products that eliminate the factors responsible for halitosis [3]. In 80-90% of cases, halitosis is due not only to poor oral hygiene and other conditions linked to the oral cavity, but also to dental problems, such as periodontitis and gingivitis [4,5]. However, there are other possible extrinsic causes, e.g. smoking, alcohol, bad diet and sociodemographic factors [6,7]. Studies performed have revealed that halitosis is due to the presence of volatile sulfur compounds (VSCs) that originate from the mouth or from the air exhaled therefrom [8-10]. Interestingly, a study on the presence of VSCs did not observe any significant differences on the prevalence of halitosis linked to gender. From this study, it therefore seems that women are more worried than men about their own oral malodor, which highlights the role of the mouth in relationships [11].

Many studies on self-reported halitosis have stressed that the problem of halitosis is often not self-perceived [6,7,12]., Few studies in the literature have highlighted the links between halitosis and emotions, e.g. anxiety [13]. Nevertheless, the relations between anxiety and halitosis have been analyzed with clinical observations suggesting that anxious situations may increase VSC concentration thus causing halitosis [14].

One specific anxious situation is dental anxiety, defined as the response to stressful dental stimuli and to dentist-patient relations [15,16]. The impact of dental anxiety on appropriate dental care would appear to be considerable [17,18].

The aim of this study is to examine the links between self-reported halitosis and factors related to emotional state, specifically dental anxiety.

## Methods

### Patients

The sample comprised 1052 subjects, 623 females and 388 males (41 subjects omitted gender) aged between 15 and 65 years old. Subjects were recruited, after giving their informed consent, in the waiting room of dental clinics of Messina and Reggio Calabria. The recruited subjects declared that they were at the dental clinic either for a first consultation or for a check-up for one of the following reasons: caries, dental cleaning, whitening, dental jewels, tartar, an abscess, dental extraction, filling, devitalization, bleeding or inflamed gums, brace, dental crown, dentures, dental surgery, pain, pyorrhea, self-reported halitosis or to accompany a patient.

Subjects agreed to fill in the protocol as a contribution to scientific research. The time spent by participants to fill in the protocol was between 90 and 120 minutes. The study project was approved by the Ethical Committee of Messina Prot. N° E392/06 (additional file n°1). Written and verbal informed consent to participate in the study was obtained from all subjects or their relative.

### Study Instruments

The protocol given to subjects was made up of the following:

1. Self-report Questionnaire to detect self-reported halitosis and other variables possibly linked to it: sociodemographic data, presence or absence of medical and dental pathologies, any allergies, oral hygiene practices, medication, smoking and alcohol consumption, the importance attributed to one's own mouth and that of others. Medical and dental pathologies were evaluated both individually and by grouping them into the following categories: for medical pathologies - gastrointestinal tract disorders: liver diseases, gastritis, ulcers; urinary system disorders: renal disease, prostate; blood diseases: anemia, rheumatic fever, other blood disorders; infections: hepatitis, sexually transmitted diseases; cardiocirculatory diseases: heart disease, heart murmur, hypertension, hypotension; respiratory diseases: emphysema, tuberculosis, asthma; diabetes; thyroid disease; skin problems; carcinoma; glaucoma; mental disorders: epilepsy, psychiatric disorders, anxiety and for dental pathologies - gingival problems; sensitive and loose teeth; bruxism; pyorrhea (additional file n°2).

2. Dental Anxiety Scale (DAS) [15,16,19] containing 19 items. This scale is divided into two parts. The first part (DAS 1) is made up of 6 items. The first five items explore the traits of dental anxiety of the patient. The replies are given using a score from 0 to 4, with a total range for this first part of between 0 and 20. The total score was considered to indicate a low anxiety level if ≤ 14 and a high level where ≥ 15.

Item 6 of DAS 1 looks at dental anxiety induced by specific dental stimuli using six sub-items: injection needles (6a), drill noise (6b), pain of treatment (6c), the smell of teeth being drilled (6d), a feeling of suffocation/gagging/lack of air (6e), the reclined position of the dentist chair (6f). Each answer is scored from 1 (most frightening) to 7 (least frightening).

The second part (DAS 2), containing 13 items, explores dental anxiety relating to dentist-patient relations. Replies are assigned a score on a descending scale from 2 to 0, with a cumulative score range of between 0 and 26. A judgment regarding the professionalism or respect for the dentist is implicit in all of the items. Anxiety level was considered to be either low (total score ≤ 12) or high (total score ≥ 13).

### Statistical analysis

Data was analyzed using the Statistical Package for Social Sciences version 16.6 [20]. Means and frequency distributions were calculated for all study variables. The chi-square test was used to examine the links between self-perceived halitosis and variables studied by the self-report questionnaire (age range, gender, level of education, occupational status, medical and dental pathologies - both singly and grouped, allergies, oral hygiene practices, medication, smoking and alcohol consumption). The mean differences between the two groups (with self-perceived halitosis and without) as regards dental anxiety (two separate subscales of DAS, specific dental fear and dentist-patient relations), and the importance attributed to one's own mouth and that of others were examined using the student's t-test. Multivariable analysis using binary logistic regression was performed to examine the importance of the various factors to the presence of self-reported halitosis in our sample. The regression model used the dependent variable of self-perceived halitosis dichotomized into "yes" or "no". The variables entered in the model, which are based on evidence in the literature about causes related to halitosis, were: relational dental anxiety (DAS 2), age > 30 years, female gender, general anxiety, poor oral hygiene, alcohol consumption, urinary system pathologies and gingival diseases.

Adjusted odds ratios and corresponding 95% confidence intervals (95% CI) were generated for all variables.

## Results

The sociodemographic characteristics of subjects are summarized in table 1.

**Table 1:** Sociodemographic characteristics of the sample.

Variables	N (%)
**Age**	
Mean	35.12
s.d.	19.38
**Sex**	
not stated	41 (3.9%)
Male	388 (36.09%)
Female	623 (59.2%)
**Education**	
not stated	385 (36.6%)
Elementary school	32 (3.0%)
Middle school	142 (13.5%)
High school graduate	317 (30.1%)
University degree	176 (16.7%)
**Occupation**	
Unemployed	386 (36.7%)
Student	193 (18.3%)
Housewife	55 (5.2%)
Manual worker	37 (3.5%)
Clerical worker	143 (13.6%)
Teacher	65 (6.2%)
Professional	120 (11.4%)
Retired	53 (5.0%)
**Self-reported Halitosis**	
yes	204 (19.39%)
no	848 (80.61%)

Total sample: N = 1052

The mean age of all participants was 35.12 years (s.d. = 19.38; range 15-65 years). Females accounted for 59.2% of the sample. As regards level of education and occupation, 30.1% of the sample had graduated from high school and 36.7% of the subjects were unemployed.

The prevalence of self-reported halitosis in this sample was 19.39% (n = 204; table 1). The sociodemographic characteristics of subjects reporting halitosis compared to the total sample are summarized in table 2. The majority of subjects reporting self-perceived halitosis fell into the following categories: age > 30 years (p < 0.001), female gender (p < 0.001), high school graduate (p < 0.050), unemployed (p < 0.001). Table 3 reports the clinical characteristics which were statistically significant for subjects with self-reported halitosis compared to the total sample: physical diseases, dental pathologies, oral hygiene practices, problems concerning stress and anxiety.

**Table 2:** Sociodemographic characteristics of subjects with self-perceived halitosis.

Variables	N (%) halitosis	N.total	**χ**^**2**^	p-value
Age				
not stated	8 (3.9%)	75 (7.1%)	129.879	p < 0.001
<30	48 (23.7%)	413 (39.4%)		
>30	108 (72.4%)	564 (53.5%)		
Gender				
not stated	36 (17.6%)	41 (3.9%)	133.387	p < 0.001
Male	79 (38.7%)	388 (36.9%)		
Female	89 (43.6%)	623 (59.2%)		
Education				
not stated	82 (40.2%)	385 (36.6%)	9.504	p < 0.050
Elementary school	10 (4.9%)	32 (3.0%)		
Middle school	32 (15.7%)	142 (13.5%)		
High school graduate	46 (22.5%)	317 (30.1%)		
University degree	34 (16.7%)	176 (16.7%)		
Occupation				
Unemployed	77 (37.7%)	386 (36.7%)	29.777	p < 0.001
Student	16 (7.8%)	193 (18.3%)		
Housewife	12 (5.9%)	55 (5.2%)		
Manual worker	13 (6.4%)	37 (3.5%)		
Clerical worker	29 (14.2%)	143 (13.6%)		
Teacher	16 (7.8%)	65 (6.2%)		
Professional	23 (11.3%)	120 (11.4%)		
Retired	18 (8.8%)	53 (5.0%)		

**Table 3:** Clinical characteristics of subjects with self-perceived halitosis.

Variables	N halitosis	N total	χ^**2**^	p-value
Anxiety	74 (36.3%)	360 (34.2%)	63.846	p < 0.001
Anxiety data missing	26 (12.7%)	38 (3.6%)		
Stress	93 (45.6%)	455 (43.3%)	57.048	p < 0.001
Smoking	61 (29.9%)	293 (27.9%)	18.371	p < 0.001
Alcohol	43 (21.1%)	182 (17.3%)	103.696	p < 0.001
Dental problems				
gum problems	124 (60.8%)	367 (34.9%)	74.726	p < 0.001
sensitive teeth	115 (56.4%)	495 (47.1%)	8.822	p < 0.005
Oral hygiene				
Yes	39 (19.1%)	310 (29.5%)	13.044	p < 0.001
No	165 (80.9%)	742 (70.5%)		
Anemia	44 (21.6%)	139 (13.2%)	43.032	p < 0.001
Thyroid	33 (16.2%)	109 (10.4%)	96.387	p < 0.001
Allergies	92 (45.1%)	443 (42.1%)	7.477	p < 0.005
Asthma	25 (12.3%)	91 (8.7%)	83.401	p < 0.001
Taking medication	82 (40.2%)	328 (31.2)	9.591	p < 0.005
Skin diseases	44 (21.6%)	133 (12.6%)	44.66	p < 0.001
Gastro-intestinal	62 (30.4%)	249 (23.7%)	89.263	p < 0.001
Urinary system	67 (32.8%)	196 (18.6%)	33.718	p < 0.001

Dental anxiety levels for the two groups of subjects (self-reported halitosis yes/no) highlighted statistically significant average differences between the two groups by reference to the two components of the scale: specific dental anxiety (DAS 1: mean = 11.00, s.d. = 4.189, t = 3.99, p < 0.001) and relational dental anxiety (DAS 2: mean = 22.05, s.d. = 6.227, t = 4.498, p < 0.001).

The analysis also looked at statistically significant differences between the two groups (self-reported halitosis yes/no) as regards the importance attributed to the one's own mouth and that of others. Differences arose for the averages relating to the importance given to the mouth of others between subjects with self-reported halitosis (mean = 6.14 and s.d. = 3.11, p < 0.001), and subjects without (mean = 7.39 and s.d. = 2.76, p < 0.001). Similarly, differences emerged for the importance attributed to one's own mouth between subjects with self-reported halitosis (mean = 6.61 and s.d. = 3.31, p < 0.001) and subjects without (mean = 8.18 and s.d. = 2.64, p < 0.001).

The logistic regression analysis results are presented in table 4. The factors most strongly linked with self-perceived halitosis are: alcohol consumption (O.R. = 0.47, p = 0.001), gingival pathologies (O.R. = 0.39, p = 0.001); age > 30 years (O.R. = 1.01, p = 0.003), urinary system pathologies (O.R. = 0.47, p = 0.003) and relational dental anxiety (DAS 2: O.R. = 1.04; p = 0.005). The other factors linked with self-perceived halitosis were: female gender (O.R. = 0.71, p = 0.041), suffering general anxiety (O.R. = 0.66, p = 0.010) and poor oral hygiene (O.R. = 0.65, p = 0.040).

**Table 4:** Logistic regression analysis of factors associated with self-reported halitosis.

Variable	β (E.S.)	O.R.	C.I.	p-value
Age > 30	0.01 (0.00)	1.01	1.00 -- 1.02	0.003
Female gender	-0.33 (0.16)	0.71	0.51 -- 0.98	0.041
DAS2	0.04 (0.01)	1.04	1.01 -- 1.07	0.005
General anxiety	-0.40 (0.15)	0.66	0.49 -- 0.90	0.010
Oral hygiene	-0.42 (0.20)	0.65	0.43 -- 0.98	0.040
Gum disease	-0.93 (0.17)	0.39	0.27 -- 0.55	0.001
Alcohol consumption	-0.73 (0.16)	0.47	0.34 -- 0.66	0.001
Urinary system	-0.75 (0.21)	0.46	0.30 -- 0.70	0.003

Significance of the model:χ^2 ^= 1034.86; p < 0.001.

## Discussion

Oral malodor seems to affect a large percentage of the general population and presents an etiology made up of several important linked factors (biological, dental, psychopathological). In our study the rate of self-reported halitosis was 19.39% and this revealed personal awareness of one's own bad breath. Nevertheless, like other studies it is possible that not all the subjects with halitosis expressly declared to be suffering from it [6,7,12]. This means perception of halitosis may differ in line with the subjectivity of perception [21]. This aspect was important in our study which evaluated the relation between the anxiety dimension and self-perceived halitosis. Moreover, the percentage of female participants (59.2%) in the sample with self-perceived halitosis poses questions on the links existing between female gender and anxiety.

Putting aside the limitations of a self-report to evaluate halitosis, we used such a scale to measure dental anxiety in our study [22]. The reliability of this scale has been demonstrated in previous studies [15,16].

The findings highlight, in line with other studies, that the etiopathogenesis of halitosis is linked to medical problems such as urinary system disorders, anemia, gastrointestinal tract disorders, skin problems, allergies, and thyroid problems (p < 0.001; table 3). Nevertheless, our study also highlighted other causes to be linked, including alcohol consumption, smoking and poor oral hygiene (p < 0.001; table 3). These data were further validated by regression analysis (table 4).

The most interesting results of this study are concerned with anxiety. Our study provides possible explanations, both biological and psychological, for the relations found between anxious situations and increased VSCs [14]. Biological, because subjects reporting halitosis are preponderantly female and they present significant associations with thyroid problems correlated in the literature with anxiety problems [23-25]; psychological, due to the declared presence of general anxiety problems (36.3%; p < 0.001; table 3) and stress (45.6%; p < 0.001; table 3).

Moreover, the specific study on the presence of dental anxiety within the group of subjects with self-reported halitosis revealed significant average differences for both subscales of dental anxiety, phobic (DAS 1: mean = 11.00, s.d. = 4.189, t = 3.99, p < 0.001) and dentist-patient relations (DAS 2: mean = 22.05, s.d. = 6.227, t = 4.498, p < 0.001). From the analysis it seems that subjects reporting halitosis were, on average, more phobic and less willing to interact with the dentist in comparison to subjects not reporting halitosis. Moreover, the regression analysis provided additional evidence as regards relational dental anxiety (DAS 2; table 4).

In addition, there were differences concerning the importance attributed to one's own mouth and that of others. Subject with self-reported halitosis on average placed less importance both on their own mouth (mean = 6.14 and s.d. = 3.11, p < 0.001) and that of others (mean = 6.61 and s.d. = 3.31, p < 0.001). This finding within the group reporting halitosis corresponds with the presence of poor oral hygiene, gingival problems and relational anxiety (referred to the dentist).

The question of which measure to use in an oral health context has been the subject of intense research efforts in recent years [26-28]. A recent study showed that good oral health has a beneficial effect on the quality of life due to its impact on appearance, breath, comfort, sleep, mood and social life [29]. Some studies have shown that dental anxiety depends on self-awareness of treatment [30,31]. In general, self-awareness is defined as the perception of oneself, and, more specifically, as the tendency to think about and evaluate aspects of oneself that are subjugated to stressful events (e.g. dental stimuli) [32,33]. This is why oral procedures are perceived as being so stressful that they can cause acute symptoms of anxiety, such as excessive apprehension, irritability, tension due to anticipated harm, and can lead to avoidance of dental treatment [34,35].

Dental fear is a common phenomenon the world over; approximately 25% of patients avoid visits and treatments, and approximately 10% reach phobic levels of anxiety [19]. This problem is of great importance for several reasons: a) avoidance causes poorer oral health and quality of life; b) high levels of anxiety and phobias may affect the dentist-patient relationship. The link between a lack of adequate dental health education and high levels of dental anxiety is important, because it causes fear in patients and poor compliance [28]. Dental anxiety relating to dentist-patient relations could be circumvented through good dental health education, regular dental visits, good patient-dentist relations and suitable communication with patients. The correlated factors of an anxiogenic perception of the dentist and self-perceived halitosis also find common ground as regards their mental representation.

It would therefore be interesting to conduct studies that draw out the consideration of others in relation to self-perception with studies including variables such as gender and ethnic group.

## Conclusions

Our study found anxiety to be one of the causes of self-reported halitosis. Halitosis therefore requires not only the professional care supplied by dentists, but also psychological support as it restricts relations with others. From this study emerges the need to promote not only healthy oral hygiene habits, but also to pay greater attention to the psychological aspects of the experience of seeing the dentist and undergoing dental treatment.

## Competing interests

The authors declare that they have no competing interests.

## Authors' contributions

SS: AT designed and coordinated the study; GT: SCG managed the statistical analysis; CM, AS: DM assisted in the conceptualization and planning of the data analysis and with manuscript preparation and review. All authors reviewed the manuscript critically for content and approved it for submission.

## Supplementary Material

Additional file 1 Ethical Committee of Messina Prot. N° E392/06 ethical notification. Click here for file

Additional file 2 Self-report Questionnaire to detect self-reported halitosis and other variables possibly linked to it. Click here for file
