# Supplementary figures and images for: Self-reported halitosis and emotional state: impact on oral conditions and treatments
Source: Health Qual Life Outcomes. 2010 Mar 26;8:34. doi: 10.1186/1477-7525-8-34 (PMC2851674; doi:10.1186/1477-7525-8-34)

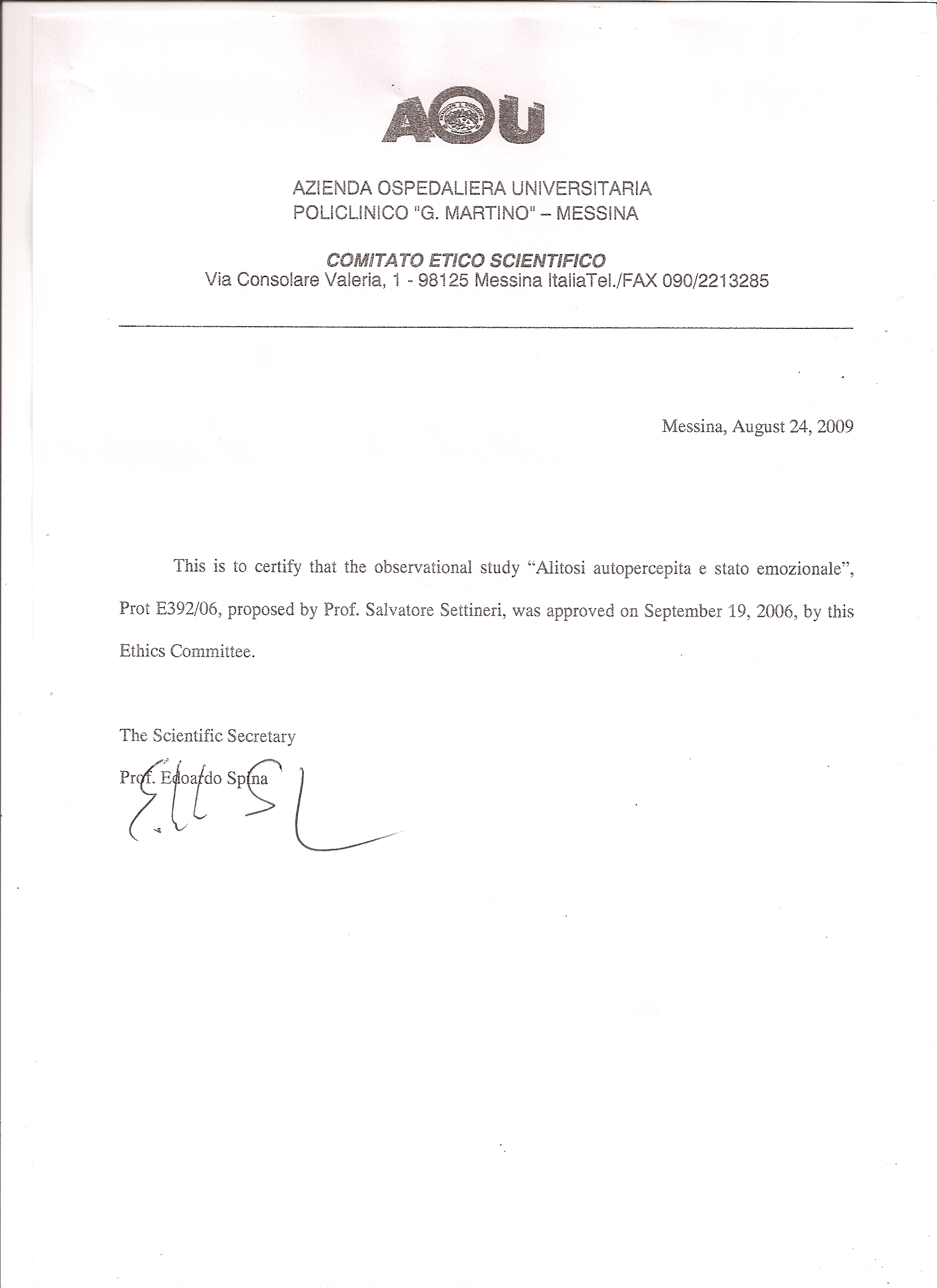

Supplement: Additional file 1 — Ethical Committee of Messina Prot. N° E392/06 ethical notification. [file 1477-7525-8-34-S1.TIFF]
