# Supplementary material for: Self-reported halitosis and emotional state: impact on oral conditions and treatments
Source: Health Qual Life Outcomes. 2010 Mar 26;8:34. doi: 10.1186/1477-7525-8-34 (PMC2851674; doi:10.1186/1477-7525-8-34)
Supplement: Additional file 2 — Self-report Questionnaire to detect self-reported halitosis and other variables possibly linked to it. [file 1477-7525-8-34-S2.DOC]

University of Messina

Degree course in dentistry

Behavioral sciences

QUESTIONNAIRE ON HALITOSIS

**Halitosis is an oral pathology caused by a number of complex factors. The questions that follow, even though they may not seem to be directly related to the mouth, can help to identify it. Therefore, all the questions are important for correct diagnosis and suitable treatment. All answers are of course strictly confidential.**

Age________________________________

Gender______________________________

Schooling____________________________

Occupation__________________________

Please answer by circling either yes or no.

1. Are you currently being treated by a doctor? YES NO

2. Are you taking any or have you recently taken any medication (e.g. tranquilisers, aspirin, cortisone)? YES NO

If yes, please specify? ______________________________________________________________

3. As far as you are aware, do you suffer from or have you ever had any of the following conditions?

Kidney disease YES NO

Thyroid problems YES NO

Liver disease YES NO

Anemia YES NO

Hepatitis YES NO

Blood disorders YES NO

Heart disease YES NO

Skin problems YES NO

Heart murmur YES NO

Swollen ankles YES NO

High blood pressure YES NO

Sexually transmitted diseases YES NO

Low blood pressure YES NO

Cancer YES NO

Rheumatic fever YES NO

Emphysema YES NO

Nosebleeds YES NO

Glaucoma YES NO

Headaches YES NO

Prostate conditions YES NO

Epilepsy YES NO

Fainting YES NO

Gastritis or ulcer YES NO

Anxiety YES NO

Mental illnesses YES NO

Tuberculosis YES NO

Asthma YES NO

Diabetes YES NO

Family relatives with diabetes YES NO

Other _____________________________________________________________________

4. Has your general state of health changed in the last year? YES NO

5. Have you gained or lost weight in the last year? YES NO

6. Have you ever had a serious illness or major surgery? YES NO

7. Have you ever had excessive bleeding after having a tooth extracted or from other wounds? YES NO

8. Have you ever suffered from allergies (to pollen, food, dust, animal fur, etc)? YES NO

9. Do you consider yourself to be anxious or stressed? YES NO

10. Do you smoke? YES NO

Please indicate what and how much you smoke ________________________________

11. Do you regularly drink alcohol? YES NO

Please state how much and what you drink _________________________________________

12. For female patients only

Are you pregnant? YES NO

Are you in menopause? YES NO

Do you take the contraceptive pill? YES NO

13. Why are you here?____________________________________________

Do you think that you suffer from:

14. bleeding gums? YES NO

15. sensitive teeth? YES NO

16. receding gums? YES NO

17. loose/wobbly teeth? YES NO

18. painful gums? YES NO

19. a tendency to grind your teeth? YES NO

20. bad breath? YES NO

21. Do you have difficulty chewing food? YES NO

22. Do you want to replace any teeth you have missing? YES NO

23. Have you ever worn braces to straighten your teeth? YES NO

24. Have you been told that you have pyorrhoea? YES NO

25. How many times a day do you clean your teeth? ______________________________________

Do you use anything else, apart from a toothbrush, to clean your teeth? __________________

26. In general, how much importance do you attach to the mouth of others? Please give a score from 1 (minimum) to 10 (maximum) where 1 means that you do not attach any importance to the mouth of other people and 10 if you attach great importance. Score: ______

27. In general, how much importance do you attach to your own mouth? Please give a score from 1 (minimum) to 10 (maximum) where 1 means that you do not attach any importance to your own mouth and 10 if you attach great importance. Score: ______
